# Vaccine Willingness and Impact of the COVID-19 Pandemic on Women’s Perinatal Experiences and Practices—A Multinational, Cross-Sectional Study Covering the First Wave of the Pandemic

**DOI:** 10.3390/ijerph18073367

**Published:** 2021-03-24

**Authors:** Michael Ceulemans, Veerle Foulon, Alice Panchaud, Ursula Winterfeld, Léo Pomar, Valentine Lambelet, Brian Cleary, Fergal O’Shaughnessy, Anneke Passier, Jonathan Luke Richardson, Karel Allegaert, Hedvig Nordeng

**Affiliations:** 1 Clinical Pharmacology and Pharmacotherapy, Department of Pharmaceutical and Pharmacological Sciences, KU Leuven, 3000 Leuven, Belgium; veerle.foulon@kuleuven.be (V.F.); karel.allegaert@kuleuven.be (K.A.); 2 Teratology Information Service, Pharmacovigilance Centre Lareb, 5237 MH ‘s Hertogenbosch, The Netherlands; a.passier@lareb.nl; 3 Service of Pharmacy, Lausanne University Hospital and University of Lausanne, 1011 Lausanne, Switzerland; alice.panchaud@chuv.ch; 4 Institute of Primary Health Care (BIHAM), University of Bern, 3012 Bern, Switzerland; 5 Swiss Teratogen Information Service, Service de Pharmacologie Clinique, Lausanne University Hospital and University of Lausanne, 1011 Lausanne, Switzerland; ursula.winterfeld@chuv.ch; 6 Materno-Fetal and Obstetrics Research Unit, Lausanne University Hospital, 1011 Lausanne, Switzerland; leo.pomar@chuv.ch (L.P.); valentine.lambelet@gmail.com (V.L.); 7 Rotunda Hospital, D01 P5W9 Dublin, Ireland; bcleary@rotunda.ie (B.C.); foshaughnessy@rotunda.ie (F.O.); 8 School of Pharmacy, Royal College of Surgeons Ireland, D02 VN51 Dublin, Ireland; 9 UK Teratology Information Service, Newcastle upon Tyne Hospitals NHS Foundation Trust, Newcastle upon Tyne NE2 4AB, UK; jonathan.richardson3@nhs.net; 10 Woman and Child, Department of Development and Regeneration, KU Leuven, 3000 Leuven, Belgium; 11 Department of Clinical Pharmacy, Erasmus MC Sophia Children’s Hospital, 3000 CA Rotterdam, The Netherlands; 12 Pharmacoepidemiology and Drug Safety Research Group, Department of Pharmacy, and PharmaTox Strategic Initiative, Faculty of Mathematics and Natural Sciences, University of Oslo, 0316 Oslo, Norway; h.m.e.nordeng@farmasi.uio.no; 13 Department of Child Health and Development, Norwegian Institute of Public Health, 0213 Oslo, Norway

**Keywords:** COVID-19, SARS-CoV-2, pregnancy, breastfeeding, counseling, maternity care, community health services, public health, primary health care, vaccine hesitancy

## Abstract

The COVID-19 pandemic may be of particular concern for pregnant and breastfeeding women. We aimed to explore their beliefs about the coronavirus and COVID-19 vaccine willingness and to assess the impact of the pandemic on perinatal experiences and practices. A multinational, cross-sectional, web-based study was performed in six European countries between April and July 2020. The anonymous survey was promoted via social media. In total, 16,063 women participated (including 6661 pregnant and 9402 breastfeeding women). Most responses were collected from Belgium (44%), Norway (18%) and the Netherlands (16%), followed by Switzerland (11%), Ireland (10%) and the UK (3%). Despite differences between countries, COVID-19 vaccine hesitancy was identified among 40–50% of the respondents at the end of the first wave of the pandemic and was higher among pregnant women. Education level and employment status were associated with vaccine hesitancy. The first wave had an adverse impact on pregnancy experiences and disrupted access to health services and breastfeeding support for many women. In the future, access to health care and support should be maintained at all times. Evidence-based and tailored information on COVID-19 vaccines should also be provided to pregnant and breastfeeding women to avoid unfounded concerns about the vaccines and to support shared decision making in this population.

## 1. Introduction

Millions of women have become pregnant, given birth and initiated breastfeeding since the start of the COVID-19 pandemic at the end of 2019 [1]. Nonetheless, the current evidence delineating the potential risks following SARS-CoV-2 infection in pregnancy still remains conflicting. While some studies concluded that pregnant women are at increased risk of severe COVID-19 including ICU admission and mechanical ventilation [2,3], other studies did not find such an association [4]. SARS-CoV-2 infection in pregnancy might also be associated with an increased risk of pregnancy complications such as preterm birth and caesarian section or could lead to vertical transmission in rare cases [5,6,7], underlining that any fetal–maternal risks of COVID-19 in pregnancy cannot be excluded. With regard to breastfeeding, there is no evidence of vertical transmission of replication-competent virus through breastmilk or that breast milk would be a source of infection for the infant [8]. In fact, SARS-CoV-2 neutralizing antibodies have been found in breastmilk of COVID-19 mothers [9]. Even in the early stages of the pandemic, mothers were advised to initiate or continue to breastfeed, even in case of suspected or confirmed COVID-19 where hygiene measures are key [10].

Over the last year, the pandemic has forced policy makers to impose containment measures to curb the transmission of the virus. Some of the measures included the obligation to work from home, the closure of schools and the abrupt discontinuation of people’s recreational and social life [11]. Although the “lockdown” had positive effects on reducing virus transmission [11,12], it may have increased mental health distress among pregnant and breastfeeding women [13,14,15,16]. In addition to the potential adverse impact on emotional wellbeing, the restrictions may also have affected women’s perinatal experiences and/or breastfeeding practices or may have been disruptive to women’s access to health services and the extent of support received [17,18]. However, pregnant and breastfeeding women do have a continuous need for medical follow-up, even in times of limited resources. Hence, insight into the impact of the pandemic on perinatal experiences and practices is vital for healthcare professionals (HCPs) and policy makers to identify consequences and challenges for perinatal care.

Since the outbreak of SARS-CoV-2, people have been eagerly anticipating the release of COVID-19 vaccine candidates to bring the pandemic to an end. Meanwhile, four vaccines have obtained market authorization in Europe (i.e., Pfizer/BioNTech, Moderna, Oxford/AstraZeneca and Johnson & Johnson vaccines) [19,20,21]. To end the pandemic, it is paramount that vaccine uptake is high enough to achieve herd immunity [22]. Vaccine coverage required for herd immunity depends on vaccine efficacy, duration of vaccine protection and the reproduction number of the virus and current estimates for SARS-CoV-2 range between 60% and 90% of the population [23]. Meanwhile, various international guidelines have been published proposing priority groups for vaccination, such as front-line HCPs, persons living in retirement facilities and patients suffering from conditions predisposing to severe disease. While the available guidelines do not recommend routine vaccination of all pregnant and breastfeeding women due to the lack of safety data, vaccinating women with a high risk of occupational exposure to the virus (e.g., health and social care personnel) or with a high risk of severe illness due to underlying conditions could be considered on an individualized basis [24,25,26,27]. However, it is still unclear whether women themselves are willing to get the vaccine during pregnancy or breastfeeding. Despite several studies on vaccine willingness in the general population [28,29], pregnant and breastfeeding women’s perceptions remain unknown.

A multinational study was conducted to explore pregnant and breastfeeding women’s beliefs about the coronavirus and COVID-19 vaccine willingness and to assess the impact of the pandemic on self-reported pregnancy and breastfeeding experiences, breastfeeding practices, access to health services and support during the breastfeeding period. Additionally, factors related to COVID-19 vaccine willingness in this perinatal cohort were explored.

## 2. Methods

### 2.1. Study Design and Sample

A multinational, cross-sectional, web-based study was performed in Ireland (IE), Norway (NO), Switzerland (CH), the Netherlands (NL) and the United Kingdom (UK) between 16 June and 14 July 2020, and in Belgium (BE) between 10 April and 31 May 2020. Pregnant and breastfeeding women up to three months postpartum (or up to 4 weeks after delivery in Belgium) and who were older than 18 years were eligible to participate. Data collection occurred through a uniform, anonymous web survey. The survey was promoted via social media and websites commonly visited by pregnant women and mothers; more information on the survey dissemination has been described elsewhere [16,17]. All participants provided an online informed consent prior to survey initiation. Ethical approval was waived in most countries as the survey was anonymous, except for Belgium (EC Research UZ/KU Leuven; S63966; 10 April 2020) and Ireland (Rotunda Hospital Research Ethics Committee; REC-2020–017; 23 June 2020). Study reporting is performed according to the STROBE guidelines (Strengthening the Reporting of Observational Studies in Epidemiology) [30].

### 2.2. Survey

The survey was part of a multinational COVID-19 research project aimed at providing insight into (1) pregnant and breastfeeding women’s mental health status during the pandemic; (2) women’s beliefs about the coronavirus and COVID-19 vaccine willingness; (3) the self-reported impact of the pandemic on the following four thematic areas of maternity care: “pregnancy and breastfeeding experiences”, “breastfeeding practices”, “access to health services” and “the extent of support during the breastfeeding period”. The findings on mental health status and the impact on the four thematic areas, as reported by the Belgian participants, have already been published [13,16,17]. The current manuscript focuses on pregnant and breastfeeding women’s beliefs about the coronavirus and COVID-19 vaccine willingness in pregnancy and breastfeeding (for all countries) and on the self-reported impact of the pandemic on the four thematic areas of maternity care (for all countries except Belgium). The procedures related to pilot testing and survey translation have been described elsewhere [16,17]. The survey was available in the official languages of the participating countries, i.e., English, German, French, Italian, Norwegian and Dutch. The pregnancy and breastfeeding survey is included in Figure S1 (Supplementary Material).

### 2.3. Measures

The surveys for pregnant and breastfeeding women were similar and were both exploratory. Information on sociodemographic characteristics was collected, including country, maternal age, relationship status, professional status, education level, smoking in pregnancy or breastfeeding. Information on the health and reproductive characteristics was also collected, including being tested for SARS-CoV-2 and the corresponding result, chronic illness, gravidity, planned pregnancy, gestational trimester, current breastfeeding duration and previous breastfeeding experience. Highest level of education attainment was categorized into low, medium or high according to national definitions. A chronic illness was considered a health condition that already existed before pregnancy.

Women’s perceptions about the coronavirus and COVID-19 vaccine willingness were assessed by seven statements (five in the pregnancy and two in the breastfeeding survey), rated on a 4-point Likert scale ranging from “strongly agree” to “strongly disagree”.

Women with a previous pregnancy or breastfeeding experience were asked to what extent the coronavirus affected their current pregnancy or breastfeeding experience, rated on a 4-point Likert scale ranging from “no influence at all” to “large influence”. With regard to breastfeeding practices, women were asked whether the pandemic had an impact on (breast)feeding patterns, along with listing reasons explaining any changes. Women who were still breastfeeding were also questioned regarding to what extent they had considered stopping breastfeeding or breastfeeding for a longer period of time due to SARS-CoV-2. These questions were rated on a 4-point Likert scale ranging from “not considered at all” to “strongly considered”. Women who indicated that the pandemic affected their access to health services or support during breastfeeding were asked how their medical follow-up (more or less follow-up or no influence) and/or support during breastfeeding (more or less support or no influence) was affected. The remaining questions examined whether women still experienced restrictions of their normal activities, as well as whether and to what extent the pandemic affected their income (negatively or positively, limited or large influence).

All participants who completed the breastfeeding survey, including those who recently stopped breastfeeding, were grouped into the category “breastfeeding women”. The breastfeeding duration was categorized into “≤6 weeks”, “between 6 weeks and 6 months” and “>6 months”.

### 2.4. Data Analysis

Descriptive statistics were used to analyze women’s sociodemographic, health and reproductive characteristics. The results of the statements on women’s beliefs about the coronavirus and COVID-19 vaccine willingness were dichotomized and tabulated, and percentages were calculated for each statement and country. Percentages were also calculated per country for the results on the four thematic areas of maternity care, including for each type of HCP (see “access to health services”) and type of support (see “support during the breastfeeding period”). Univariable and multivariable logistic regression was performed to identify factors related to COVID-19 vaccine willingness with regard to pregnant and breastfeeding women’s sociodemographic, health and reproductive characteristics. Results were shown as crude (OR) and adjusted odds ratios (aOR) and 95% confidence intervals (CI). All sociodemographic, health and reproductive characteristics were used in the univariable analyses. Only significant variables (*p* ≤ 0.05) were included in the adjusted models, such as country, maternal age, professional status, highest education level, gravidity, current breastfeeding duration and previous breastfeeding experience. Multicollinearity of the variables was checked using chi-square tests. The variable “chronic illness” was not addressed in the Belgian survey and was therefore not included in the calculation of the adjusted models. All survey responses, including those with incomplete results, were included in the analysis. Data analysis was performed using SPSS Statistics version 26 (IBM Corp, Armonk, NY, USA).

## 3. Results

### 3.1. Characteristics of the Participants

In total, 16 063 women participated in the study (i.e., 6661 pregnant and 9402 breastfeeding women) (see Table 1). Most responses were collected from Belgium (44%), Norway (18%) and the Netherlands (16%), followed by Switzerland (11%), Ireland (10%) and the UK (3%). More than half of the pregnant women were in their third trimester (51%), while 91% of the mothers were still breastfeeding at the time of survey completion. Most breastfed infants were between 6 weeks and 6 months old (44%). A chronic illness was reported by 19% and 16% of the pregnant and breastfeeding women, respectively. With regard to COVID-19, less than 1% had tested positive for SARS-CoV-2. One-third of the professionally active respondents were employed in healthcare (35%). A comparison of the sociodemographic characteristics of the participants with country-specific birthing population data is shown in Table S1 (Supplementary Material). Differences related to relationship status, professional status, education attainment and smoking in pregnancy.

### 3.2. Beliefs about the Coronavirus and COVID-19 Vaccine Willingness

While 54% of the pregnant women agreed that a coronavirus infection during pregnancy could affect the development of the unborn child, 86% believed that this could be possible in cases of severe COVID-19 in pregnancy. Nonetheless, 96% of the pregnant women would not consider a termination of pregnancy in case of a SARS-CoV-2 infection in the first trimester.

At the time of survey completion, 61% of the pregnant respondents answered that they would be willing to get vaccinated against SARS-CoV-2 in pregnancy if a vaccine was available (see Table 2). If Belgium was excluded from the sample (i.e., because the data were collected at a different time), the proportion of women likely to be administered a COVID-19 vaccine in pregnancy dropped to 49%. With regard to breastfeeding women, 69% was willing to get vaccinated against SARS-CoV-2 while breastfeeding if a vaccine was available (see Table 2). If Belgium was again excluded from the sample, the percentage dropped to 59%. Although French-speaking participants were only a small part of the Belgian sample (11–13%), they were less likely to get a vaccine during pregnancy (49%) or breastfeeding (55%) compared to their Dutch-speaking counterparts (83% and 82%, respectively). Finally, 86% and 81% of all pregnant and breastfeeding women were not willing to participate in a scientific study testing medicines against COVID-19. A detailed overview of the country-specific results is included in Table S2 (Supplementary Material).

With regard to factors related to vaccine willingness (see Table 3), a higher likelihood of getting a COVID-19 vaccine during pregnancy or breastfeeding was observed among women living in Belgium (measured in April–May). Primigravida pregnant women and breastfeeding women who delivered in the last 6 months were also more likely to be willing to get a vaccine. In contrast, pregnant and breastfeeding women with low and medium levels of education and without employment were less in favor of COVID-19 vaccination. Finally, pregnant women working in healthcare were also less likely to be willing to get a vaccine compared to women employed outside healthcare, although the difference in absolute numbers was very limited (62% vs. 65%, see Supplementary Material Table S3).

In the univariable analyses, pregnant women who had a positive test result for SARS-CoV-2 were more in favor of getting a COVID-19 vaccine in pregnancy compared to women without a positive test result, but the finding was not significant (OR 1.65; 95% CI: 0.80–3.43). A similar observation was noted among breastfeeding women (OR 1.23; 95% CI: 0.67–2.28).

### 3.3. Impact on Pregnancy and Breastfeeding Experiences

In total, 52% of those who had already been pregnant (*n* = 2044) reported that the pandemic had a (rather) large impact on their current pregnancy experience compared to previous pregnancies (UK: 75%; IE: 71%; NO: 64%; CH: 37%; NL: 34%). Main reasons negatively affecting the current pregnancy experience were the absence of the partner during antenatal check-ups and ultrasounds, less medical follow-up, increased anxiety or stress due to the current situation, social isolation, less support, as well as being more cautious when interacting with other people (see Table S4 in the Supplementary Material for a list of representative statements). In contrast, only 17% of the women with a previous breastfeeding experience (*n* = 2786) indicated that the pandemic had a (rather) large impact on how they dealt with breastfeeding (IE: 32%; UK: 28%; NO: 17%; NL: 9%; CH: 9%).

### 3.4. Impact on Breastfeeding Practices

Overall, 96% of the women (*n* = 4623) indicated that the infant’s diet had not changed due to the pandemic (CH: 98%; NO: 98%; NL: 96%; IE: 93%; UK: 92%). However, in cases where the mother indicated the diet had changed due to the pandemic (*n* = 164), infants more often received mother’s milk in 73% of the cases as compared to before the coronavirus outbreak. Moreover, 90% of the mothers who quit breastfeeding in the preceding three months (*n* = 446) reported that breastfeeding cessation was not the result of the pandemic (NO: 95%; CH: 95%; NL: 92%; UK: 92%; IE: 81%). Main reasons for breastfeeding cessation or decline were insufficient support from an HCP with regard to breastfeeding and/or related issues, and other childcare responsibilities or circumstances at home. Main reasons for an increase in giving breastmilk were that being or working from home during the pandemic facilitated breastfeeding, and women’s beliefs to protect the infant against the coronavirus through mother’s milk (see Supplementary Material Table S4). Finally, 96% of the women (*n* = 4576) had not yet considered (at all) stopping giving breastmilk because of the coronavirus (NL: 98%; NO: 98%; CH: 98%; UK: 89%; IE: 89%). In fact, 49% had already (strongly) considered giving breastmilk for a longer period of time because of the coronavirus (IE: 60%; NO: 49%; UK: 48%; CH: 47%; NL: 44%).

### 3.5. Impact on Access to Health Services

Overall, 59% of the pregnant women (*n* = 3844) cited that the pandemic affected their access to health services to some extent (UK: 93%; IE: 79%; NL: 59%; NO: 51%; CH: 48%). Women mainly reported less medical follow-up by midwives (67%), general practitioners (GPs) (51%) and obstetricians (44%) (see Table 4). In each country, less than 10% reported having received more medical follow-up from any type of HCP.

Of all breastfeeding women (*n* = 4865), 54% felt that their access to health services during the breastfeeding period was affected by the pandemic (UK: 86%; IE: 86%; NO: 70%; CH: 31%; NL: 30%). Women mainly reported less medical follow-up by perinatal organizations (72%), lactation consultants (69%), GPs (67%) and midwives (66%) (see Table 4). In each country, less than 10% reported having received more medical follow-up from any type of HCP. An overview of the routine antenatal care in the countries along with the COVID-19 regulations in maternity care during the pandemic is provided in Figure S2 (Supplementary Material). The country-specific results of the self-reported impact of the pandemic on access to health services are included in Table S5 and Table S6 (Supplementary Material).

### 3.6. Impact on Support during the Breastfeeding Period

Overall, 42% of the breastfeeding women (*n* = 4817) indicated that the pandemic affected the support they received during the breastfeeding period to some extent (IE: 74%; UK: 74%; NO: 42%; CH: 36%; NL: 24%). Women mainly reported less support from friends (81%), perinatal organizations (81%) and maternity care services at home (76%) (see Table 5). In each country, a maximum of 15% reported receiving more of any type of support compared to before the pandemic. An overview of the country-specific results of the self-reported impact of the pandemic on support during breastfeeding is included in Table S7 (Supplementary Material).

### 3.7. Impact on Women’s Personal Life and Financial Situation

At the time of survey completion, 81% of the pregnant women (*n* = 3626) indicated that their normal activities were (still) restricted as compared to before the pandemic (UK: 95%; IE: 94%; NO: 87%; CH: 69%; NL: 68%); this was the case for 82% of the breastfeeding women (*n* = 4644) (UK: 97%; IE: 95%; NO: 92%; NL: 73%; CH: 69%). In general, 70% of the pregnant women indicated having personally restricted themselves to only essential journeys during the pandemic on top of the measures imposed by the government (IE: 86%; UK: 83%; CH: 69%; NO: 69%; NL: 60%). The corresponding percentage for breastfeeding women was 57% (IE: 66%; NL: 59%; NO: 58%; UK: 52%; CH: 45%).

With regard to women’s financial situation, 22% of the pregnant women (*n* = 3129) cited that the pandemic affected their professional income to some extent (UK: 40%; IE: 32%; NO: 23%; CH: 21%; NL: 14%), with a predominantly negative (90%) but limited (58%) impact noted across countries. Likewise, 18% of the breastfeeding women (*n* = 3842) felt that the pandemic affected their professional income (UK: 25%; IE: 24%; NO: 20%; CH: 16%; NL: 12%). In this group, a negative (89%) but limited (58%) effect was also reported.

## 4. Discussion

### 4.1. Main Findings

This multinational study aimed to explore pregnant and breastfeeding women’s beliefs about the coronavirus and COVID-19 vaccine willingness and to assess the impact of the pandemic on self-reported perinatal experiences and practices. A uniform, cross-sectional web survey was distributed across six European countries during the first wave of the pandemic (April–July 2020) [31], capturing in total more than 16,000 unique responses including statements reflecting women’s experiences and practices throughout the pandemic. To the best of our knowledge, this is the largest study reported so far exploring COVID-19 vaccine willingness in a perinatal population.

Overall, about 60–70% of the women indicated that they were willing to get a COVID-19 vaccine during pregnancy or breastfeeding; all countries had a larger proportion of breastfeeding women in favor of the vaccine. However, and as previously shown [32,33], substantial differences were observed across countries (range: 30–80%), and even between communities within the same country (Belgium, range: 49–82%). Differences between countries may be, at least partially, explained by the different timing of study execution and consequently by the different stage of the pandemic with different incidence rates of COVID-19 cases and containment measures in place at the time of survey completion (Belgium = April–May 2020, i.e., at the peak of the first wave of the pandemic when a strict lockdown was still in effect; other countries = June–July 2020, i.e., at the end of the first wave of the pandemic in most countries, except the UK, when restrictions were mostly lifted) (see Supplementary Material Table S8 and Figure S3) [31]. This hypothesis is supported by two recent longitudinal studies on the public’s likelihood of getting a COVID-19 vaccine, showing a decline of 17% in vaccine willingness between April and September 2020 [29,32]. A recent large-scale modeling study also acknowledged that vaccine confidence can vary over time and across countries [34]. In our sample, the percentages observed during the first wave of the pandemic (i.e., in Belgium, 79%) are in line with estimates calculated at that time in other samples within the general public in Europe and America (74%) [29,35]. When assessing our data collected at the end of the first wave (i.e., in all countries except Belgium), vaccine willingness dropped to 50–60% of pregnant and breastfeeding women. For breastfeeding women, the observed likelihood of getting a vaccine whilst breastfeeding (59%) is quite similar to estimates measured within the general public in European (68%) and American samples (62%) at that time [29,32]. However, COVID-19 vaccine willingness during pregnancy, as measured in our sample (49% if excluding Belgian data), is lower than observed in the other samples at that time (62–68%) [29,32], but in line with estimates calculated among an international cohort of pregnant women in October–November 2020 (52%) [33]. Thus, our findings do not only demonstrate that COVID-19 “vaccine hesitancy” is also prevalent in the perinatal population, but that it might occur even more often in pregnancy. This would not be entirely surprising as previous studies have shown that women do have a higher threshold to use medicines during pregnancy [36,37,38,39]. Finally, the low vaccine willingness among Swiss women is remarkable and might be explained by the higher percentage of low educated women in the Swiss cohort and the lower proportion of Swiss breastfeeding participants who were professionally active. This should be further explored in future studies.

The lack of safety data on COVID-19 vaccines in pregnancy and breastfeeding increases gender-based health inequalities across the world [40,41]. Although current guidelines do not recommend routine vaccination of pregnant and breastfeeding women, vaccinating women with a high risk of exposure to SARS-CoV-2 or with underlying conditions may be considered on an individualized basis [24,25,26,27]. Based on our findings, specific attention should be given in clinical practice to low(er) educated and unemployed women as they appeared to be less willing to get the vaccine. This is in line with previous studies showing the increased risk of COVID-19 vaccine hesitancy among low(er) educated patients [28,29,32], which should alert HCPs and policy makers involved in vaccination recommendations given its high public health importance. However, further research is still needed to ascertain which subgroups in the perinatal population may have a higher likelihood of COVID-19 vaccine hesitancy.

While half of the pregnant women indicated that the pandemic affected their ongoing pregnancy experience, only a minority of breastfeeding women felt that COVID-19 had an impact on how they personally dealt with breastfeeding. As reflected by the quotations and described elsewhere [42,43], pregnant women attributed their negative experience to the reduced medical follow-up, the absence of the partner during antenatal care visits and increased anxiety or stress due to the current situation. Overall, breastfeeding women generally denied that the pandemic influenced their breastfeeding practices. In fact, very positive beliefs about the importance of breastfeeding in times of COVID-19 were observed, in line with the findings of the recent study in Belgium [17]. However, breastfeeding women also reported disruptions to medical and social support services, as shown earlier [18,44]. It cannot be excluded that insufficient support from an HCP or other childcare responsibilities at home led to premature breastfeeding cessation in individual cases. Therefore, HCPs are advised to consider the patient-specific context or home situation when counseling breastfeeding women in the future.

### 4.2. Strengths and Limitations

This multinational study was initiated within the European Network of Teratology Information Services (ENTIS). The participation of six countries resulted in a large sample size (>16,000 women). A uniform data collection instrument was used across all countries at the same time (except Belgium), allowing for a comparison of the findings between countries. The findings on vaccine willingness are among the first to be reported in a perinatal population and convey an important aspect that should not be neglected in the ongoing debate on COVID-19 vaccination guidance in pregnancy and breastfeeding. The findings on perinatal experiences and practices, including women’s quotations, provide salient evidence on the impact of the pandemic, thereby identifying opportunities for the organization of perinatal care during and in the wake of the pandemic.

Some limitations should also be considered regarding the validity of the findings. First, our findings on COVID-19 vaccine willingness should be interpreted in light of the fact that no vaccine candidates were approved or under review in Europe at the time of survey completion, and that the data were collected before the first reports on the efficacy of COVID-19 vaccine candidates were published. Official recommendations on the vaccination of pregnant and breastfeeding women were also not available at that time yet. It is reasonable to assume that such events could influence people’s perceptions. Due to the overall lack of published data on COVID-19 vaccine willingness in the perinatal population, we could basically only compare our estimates with data obtained from the general public. The different timing of survey completion across countries along with the different stage of the pandemic and restrictions in place further impede the generalizability of the findings. Second, an online survey using self-reported measures was applied, entailing the risk of selection bias. Overall, participants were more frequently in a relationship, higher educated, professionally active (often in healthcare) and non-smokers compared to the general birthing population in each country [16]. As women with a low(er) education attainment and without employment tend to be less likely to get vaccinated [28,29,32], the percentage of vaccine willingness observed in our perinatal sample might be an overestimation of the actual situation. This could, for example, mask evidence of a higher threshold for COVID-19 vaccination among pregnant and breastfeeding women. The large proportion of women who were breastfeeding for > 6 months might have contributed to the overall lack of self-reported impact of the pandemic on breastfeeding experiences and practices as well as the positive perceptions towards breastfeeding in our sample. However, as the breastfeeding experiences were so predominantly positive, we believe they reflect the actual situation. The cross-sectional study design and the lack of longitudinal data collection further prevented us from drawing conclusions on temporal changes in vaccine willingness and on the impact of COVID-19 on the initiation and total duration of breastfeeding during the pandemic. Third, neither qualitative data on potential reasons explaining vaccine hesitancy nor data on the type of information women need about COVID-19 vaccines or how to deliver it were collected. The specific reason for SARS-CoV-2 testing of individual women also remained unknown, as well as information on race, ethnicity or socioeconomic status. Lastly, only a few UK residents participated in this study, thus requiring careful interpretation of the UK findings.

### 4.3. Future Perspectives

The specific timing when this study was performed, covering the first wave of the pandemic, should be considered when interpreting the findings on vaccine willingness. Future studies are needed to investigate pregnant and breastfeeding women’s vaccine willingness at a later stage of the pandemic, at a time when COVID-19 vaccines are being given to the general public on a large scale. Moreover, it would be interesting to assess whether pregnant women indeed have a higher threshold for COVID-19 vaccination compared to their breastfeeding counterparts. Equally important, and to optimally tackle COVID-19 vaccine hesitancy, potential drivers and barriers of vaccine confidence among pregnant and breastfeeding women could be explored, as well as the link between vaccine confidence and uptake in these groups. Knowledge about determinants of vaccine distrust and acceptance will allow the development of relevant interventions (e.g., information campaigns) aimed at increasing vaccine coverage, especially in high-risk patients [45].

The exclusion of the perinatal population from COVID-19 vaccine trials, and consequently, the lack of safety data of vaccine candidates is challenging for clinical practice, especially given that so many front-line healthcare workers are women of reproductive age [40,41]. Recently, COVID-19 surveillance systems have been set up in multiple countries and are imperative to closely monitor the utilization and safety of COVID-19 vaccines and different vaccine platforms in pregnant and breastfeeding women [46]. More safety data will allow HCPs to provide reliable, evidence-based and uniform information to patients in case of, for example, unplanned pregnancies after vaccination.

Finally, the pandemic reduced women’s contact with HCPs and support services. It is likely that the pandemic accelerated the wide usage of telemedicine [47]. However, to guarantee successful implementation, it would be pivotal to investigate how patients and HCPs perceive technology-assisted medicine and the usage of mobile health applications for remote reproductive health care.

## 5. Conclusions

Despite differences across countries, COVID-19 vaccine hesitancy was identified among 40–50% of the pregnant and breastfeeding women at the end of the first wave of the pandemic and was higher among pregnant women. Education level and employment status were associated with vaccine hesitancy. The first wave had an adverse impact on pregnancy experiences and disrupted access to health services and breastfeeding support for many women. However, the pandemic stimulated many women to continue breastfeeding, although premature breastfeeding cessation due to insufficient support cannot be excluded in some cases. In the future, access to health care and support should be maintained at all times. Evidence-based and tailored information on the safety and efficacy of COVID-19 vaccines should also be provided to pregnant and breastfeeding women to avoid unfounded concerns about the vaccines and to support shared decision making in this population.

## Figures and Tables

**Table 1:** Overview of the characteristics of the participants (*n* = 16 063).

	Pregnant Women (*n* = 6 661)	Breastfeeding Women (*n* = 9 402)
	% (*n*)	% (*n*)
**Sociodemographic characteristics**		
**Country**		
Belgium	41.3 (2754)	45.4 (4268)
Ireland	10.4 (692)	9.7 (912)
Norway	20.2 (1344)	15.8 (1485)
Switzerland	8.5 (563)	12.7 (1193)
The Netherlands	17.6 (1173)	15.4 (1447)
United Kingdom	2.0 (135)	1.0 (97)
**Maternal age (years)**		
18–25	6.2 (373)	3.2 (266)
26–30	36.7 (2191)	29.1 (2436)
31–35	42.1 (2515)	46.7 (3907)
36–40	13.5 (806)	18.0 (1504)
>40	0.1 (88)	3.0 (254)
**Relationship status**		
Partner	97.8 (5868)	97.7 (8213)
No partner	2.2 (132)	2.3 (191)
**Professional status**		
Professionally active, not in healthcare	59.3 (3499)	58.4 (4837)
Professionally active, in healthcare	33.0 (1950)	31.6 (2618)
Not professionally active	7.7 (454)	10.0 (826)
**Education level**		
Low	3.2 (190)	3.9 (319)
Medium	20.6 (1217)	21.0 (1734)
High	76.2 (4494)	75.1 (6194)
**Smoking in pregnancy/breastfeeding**		
Yes	2.5 (151)	3.5 (296)
No	97.5 (5849)	96.5 (8110)
Health and reproductive characteristics		
**SARS-CoV-2**		
Tested	6.5 (421)	8.9 (803)
Tested positive	0.6 (38)	0.6 (52)
**Chronic illness^a^**		
Yes	19.2 (623)	16.1 (665)
No	80.8 (2623)	83.9 (3473)
**Gravidity**		
Primigravida	47.8 (3183)	N/A
Multigravida	52.2 (3478)	N/A
**Planned pregnancy**		
Yes	85.4 (5690)	N/A
No	14.6 (971)	N/A
**Gestational trimester**		
First trimester (0–12 weeks)	9.9 (653)	N/A
Second trimester (13–27 weeks)	38.9 (2558)	N/A
Third trimester (28–40 weeks)	51.1 (3357)	N/A
**Current breastfeeding duration**		
≤6 weeks	N/A	17.5 (1512)
Between 6 weeks–6 months	N/A	43.6 (3758)
>6 months	N/A	38.9 (3357)
**Previous breastfeeding experience**		
Yes	N/A	55.1 (5113)
No	N/A	44.9 (4173)

Numbers may not add up due to missing values. ^a^ information about the variable “chronic illness” was not available for Belgian participants. N/A: not applicable.

**Table 2:** COVID-19 vaccine willingness among pregnant and breastfeeding women.

Statement	(Strongly) Agree						
	Total	BE	NO	UK	IE	NL	CH
“If a coronavirus vaccine was available, I would get the vaccine during pregnancy.” (*n* = 6420)	61.4%	78.1%	55.1%	53.9%	51.1%	48.6%	29.7%
	−3943	−2150	−741	−62	−316	−521	−153
“If a coronavirus vaccine was available, I would get the vaccine during breastfeeding.” (*n* = 8980)	68.8%	79.2%	67.6%	79.7%	67.1%	60.4%	38.6%
	−6174	−3379	−1004	−63	−536	−793	−399

Results are expressed as % (absolute numbers). The statements were rated on a 4-point Likert scale ranging from (strongly) agree to (strongly) disagree. Only the percentages of women (strongly) agreeing are shown in the table. BE = Belgium; NO = Norway; UK = United Kingdom; IE = Ireland; NL = the Netherlands; CH = Switzerland.

**Table 3:** Factors related to COVID-19 vaccine willingness among pregnant and breastfeeding women.

Characteristic	Pregnant Women		Breastfeeding Women	
	OR * (95% CI)	aOR ** (95% CI)	OR * (95% CI)	aOR *** (95% CI)
*Country*				
Belgium	Ref	Ref	Ref	Ref
Ireland	0.29 (0.24–0.35)	**0.33 (0.27–0.40)**	0.54 (0.46–0.63)	**0.60 (0.49–0.74)**
Norway	0.35 (0.30–0.40)	**0.35 (0.30–0.41)**	0.55 (0.48–0.63)	**0.54 (0.47–0.63)**
Switzerland	0.12 (0.10–0.15)	**0.15 (0.11–0.19)**	0.17 (0.14–0.19)	**0.20 (0.17–0.25)**
The Netherlands	0.27 (0.23–0.31)	**0.29 (0.25–0.34)**	0.40 (0.35–0.46)	**0.42 (0.36–0.49)**
United Kingdom	0.33 (0.23–0.48)	**0.32 (0.21–0.49)**	1.04 (0.60–1.80)	1.16 (0.60–2.26)
*Maternal age (years)*				
18–25	Ref	Ref	Ref	Ref
26–30	1.41 (1.13–1.76)	0.94 (0.73–1.20)	1.51 (1.17–1.96)	1.15 (0.84–1.57)
31–35	1.47 (1.18–1.83)	1.05 (0.81–1.35)	1.60 (1.24–2.06)	1.27 (0.93–1.73)
36–40	1.21 (0.95–1.55)	1.05 (0.79–1.40)	1.60 (1.22–2.10)	**1.44 (1.04–2.01)**
>40	0.89 (0.56–1.41)	0.76 (0.45–1.28)	1.27 (0.89–1.82)	1.23 (0.79–1.89)
*Professional status*				
Active, but not in healthcare	Ref	Ref	Ref	Ref
Active in healthcare	0.90 (0.80–1.01)	**0.84 (0.74–0.95)**	0.95 (0.85–1.05)	0.91 (0.81–1.03)
Not professionally active	0.55 (0.45–0.67)	**0.71 (0.57–0.89)**	0.51 (0.44–0.60)	**0.69 (0.57–0.82)**
*Highest education level*				
Low	0.26 (0.19–0.35)	**0.58 (0.41–0.81)**	0.23 (0.18–0.28)	**0.54 (0.41–0.73)**
Medium	0.57 (0.50–0.64)	**0.71 (0.62–0.83)**	0.60 (0.54–0.68)	**0.68 (0.59–0.78)**
High	Ref	Ref	Ref	Ref
*Chronic illness*				
Yes	0.99 (0.83–1.18)	N/A	1.25 (1.05–1.49)	N/A
*Gravidity*				
Primigravida	1.15 (1.04–1.27)	**1.16 (1.03–1.31)**	N/A	N/A
*Current breastfeeding duration*				
≤6 weeks	N/A	N/A	1.84 (1.58–2.13)	**1.73 (1.46–2.05)**
Between 6 weeks–6 months	N/A	N/A	1.29 (1.16–1.44)	**1.40 (1.24–1.58)**
>6 months	N/A	N/A	Ref	Ref
*Previous breastfeeding experience*				
Yes	N/A	N/A	0.90 (0.82–0.98)	0.92 (0.82–1.04)

N/A = not available; * OR = crude odds ratio; ** aOR = adjusted odds ratio, adjusted for country, maternal age, professional status, highest education level and gravidity. *** aOR adjusted for country, maternal age, professional status, highest education level, current breastfeeding duration and previous breastfeeding experience. The bold numbers indicate aORs not including 1. Only significant variables were included in the adjusted models and are shown in the table. Information on “chronic illness” was not available for Belgian respondents and was therefore not included in the adjusted models. Reference groups for the variables “chronic illness”, “gravidity” and “previous breastfeeding experience” were no chronic illness, multigravida and no previous breastfeeding experience, respectively.

**Table 4:** Impact of the pandemic on access to health services during pregnancy and lactation (*n* = 8709).

Healthcare Professional	More Follow-Up		Less Follow-Up		No Influence	
	Pregnancy	Lactation	Pregnancy	Lactation	Pregnancy	Lactation
Midwife	4.9% (93)	5.2% (95)	67.3% (1290)	65.9% (1206)	27.8% (533)	28.9% (530)
General practitioner	6.5% (98)	3.4% (71)	50.6% (764)	66.5% (1381)	42.9% (648)	30.1% (624)
Obstetrician	6.1% (72)	2.9% (40)	43.5% (515)	43.3% (588)	50.4% (597)	53.7% (729)
Medical specialist	7.7% (66)	3.0% (30)	36.5% (313)	47.5% (482)	55.8% (479)	49.5% (502)
Perinatal organization*	N/A	3.3% (37)	N/A	72.0% (811)	N/A	24.8% (279)
Lactation consultant	N/A	3.1% (55)	N/A	69.0% (1230)	N/A	27.9% (497)
Pediatrician	N/A	3.5% (57)	N/A	38.1% (612)	N/A	58.3% (937)

N/A = not applicable. Results are expressed as % (absolute numbers). Percentages were calculated using as denominator the total number of women who reported that their access to health services was affected by the pandemic and who indicated being counseled by this type of professional. * The variable “perinatal organization” was not included in the Norwegian survey.

**Table 5:** Impact of the pandemic on the extent of support during breastfeeding (*n* = 2008).

Support Provided by	More Support	Less Support	No Influence
Friends	5.4% (101)	81.3% (1535)	13.3% (251)
Perinatal organization	3.1% (45)	80.5% (1165)	16.4% (237)
Maternity care services at home	3.4% (57)	75.8% (1273)	20.8% (350)
Family	11.6% (224)	73.5% (1415)	14.9% (286)

Results are expressed as % (absolute numbers). Percentages were calculated using as denominator the total number of women who reported that the support they received during the breastfeeding period was affected by the pandemic and who indicated that this type of support was applicable to them during breastfeeding.

## Data Availability

The data presented in this study are available in the article and Supplementary Materials.

## Supplementary Materials

The following are available online at https://www.mdpi.com/1660-4601/18/7/3367/s1, Figure S1: The short English version of the pregnancy and breastfeeding survey; Table S1: Comparison of the sociodemographic characteristics of the participants with country-specific birthing population data; Table S2: Country-specific results of pregnant and breastfeeding women’s perceptions about the coronavirus; Table S3: Country-specific results of COVID-19 vaccine willingness during pregnancy and breastfeeding according to professional status; Table S4: Overview of representative statements of participants; Figure S2: Overview of routine antenatal care in participating countries along with the COVID-19 regulations regarding maternity care; Table S5: Country-specific results of pregnant women’s self-reported impact of the pandemic on access to health services; Table S6: Country-specific results of breastfeeding women’s self-reported impact of the pandemic on access to health services; Table S7: Country-specific results of breastfeeding women’s self-reported impact of the pandemic on support during breastfeeding. Table S8: Imposed regulations per country during the first wave of the COVID-19 pandemic. Figure S3: Overview of the COVID-19 cases and death notification rates per country during the first wave of the pandemic.
