# Supplementary material for: Vaccine Willingness and Impact of the COVID-19 Pandemic on Women’s Perinatal Experiences and Practices—A Multinational, Cross-Sectional Study Covering the First Wave of the Pandemic
Source: Int J Environ Res Public Health. 2021 Mar 24;18(7):3367. doi: 10.3390/ijerph18073367 (PMC8038007; doi:10.3390/ijerph18073367)

Supplementary Material

Figure S1: The short English version of the pregnancy and breastfeeding survey

1. How did you hear about this study?
   a. Via social media
   b. Via word of mouth
   c. Via email
   d. I received a call
   e. I received a text message
   f. Other:……………………………………..

2. Are you 18 years or older?
   a. Yes
   b. No
   If no, end of survey. Thank you for your time.

3. Please indicate which describes your situation right now. In case you are pregnant and breastfeeding at the same time, please select the option "I breastfed in the last 3 months". A specific question in the latter survey will ask you about your current situation.
   a. I am currently pregnant
   b. I breastfed in the last 3 months
   c. None of the above situations
   End of survey. Thank you for your time.

YOUR PREGNANCY

1. How many weeks pregnant are you right now? (open answer)
2. Was the pregnancy planned?
    a. Yes
    b. No
    c. No, but it was not unexpected

3. Have you been pregnant before?
    a. Yes
    b. No

4. If yes: How many children do you already have (not including the current pregnancy) (write your answer down <u>in numbers)?</u>

5. If yes, compared with your previous pregnanc(y)(ies), to what extent has the coronavirus affected your experience of your current pregnancy?
    a. No influence at all
    b. Rather no influence
    c. Rather large influence
    d. Large influence

6. Has the coronavirus pandemic affected how you interact with health services during your current pregnancy?
    a. Yes
    b. No

7. If yes, please indicate how your interaction with health services has been affected by the coronavirus pandemic, and whether you receive more, less or equal follow-up as before (more follow-up, less follow-up, no influence, not applicable)
    a. Follow-up by the obstetrician
    b. Follow-up by the midwife
    c. Follow-up by the general practitioner
    d. Follow-up by the specialist

CORONAVIRUS SYMPTOMS

8. Did you receive a test to determine coronavirus infection?
    a. Yes
    b. No

9. If yes: Did you receive a nose/throat swab?
    a. Yes
    b. No

10. If yes: What was the result of the test? If you received multiple tests, please indicate 'positive' if at least one of the test results was positive
    a. Positive (infected with the coronavirus)
    b. Negative (not infected with the coronavirus)
    c. I don't know (yet)

11. If yes: Did you receive a blood test?
    a. Yes
    b. No

12. If yes: What was the result of the test?
    a. Confirmation of a current coronavirus infection
    b. Confirmation of a previous coronavirus infection
    c. No coronavirus infection
    d. I don't know (yet)

13. If yes: Did you receive a CT-scan of the lungs to determine a coronavirus related pneumonia?
    a. Yes
    b. No

14. If yes: what was the result of the test?
    a. The scan determined a coronavirus infection
    b. The scan did not determine a coronavirus infection
    c. I don’t know (yet)

PERSONAL BELIEFS ABOUT THE CORONAVIRUS AND COVID-19 VACCINES

Please indicate to which extent you agree with the following statements:

15. I believe that a coronavirus infection during pregnancy can affect the development of the unborn child.
    a. Strongly agree
    b. Agree
    c. Disagree
    d. Strongly disagree

16. I believe that a coronavirus infection which seriously affects the health of the mother can affect the development of the unborn child.
    a. Strongly agree
    b. Agree
    c. Disagree
    d. Strongly disagree

17. I would consider a termination of pregnancy in case of a coronavirus infection during the first trimester of pregnancy.
    a. Strongly agree
    b. Agree
    c. Disagree
    d. Strongly disagree

18. If a coronavirus vaccine was available, I would get the vaccine during pregnancy.
    a. Strongly agree
    b. Agree
    c. Disagree
    d. Strongly disagree

19. If I was infected with coronavirus during pregnancy, I would be willing to participating in a scientific study where experimental medicines are tested.
    a. Strongly agree
    b. Agree
    c. Disagree
    d. Strongly disagree

CORONAVIRUS AND RESTRICTED ACTIVITIES

20. Did you personally decide to restrict yourself to only essential journeys during the corona pandemic (on top of the measures imposed by the government) due to your pregnancy?
    a. Yes
    b. No

21. Are your normal activities currently restricted compared to before the coronavirus period?
    a. Yes
    b. No

PERSONAL BACKGROUND
In this final part, we will ask you a few questions about yourself as a participant of this study.

About you

22. Year of birth?

23. Where do you live?

24. Do you currently have a partner?
    a. Yes
    b. No

25. Have you smoked in the past 4 weeks?
    a. Yes
    b. No

26. Do you have any chronic illnesses (these are conditions that already existed before your pregnancy)?
    a. Yes
    b. No

27. If yes: which chronic illness(es) do you have?
    a. Asthma
    b. Allergy
    c. Cardiovascular disease (including high blood pressure, high cholesterol, heart disease,..)
    d. Depression
    e. Diabetes
    f. Epilepsy
    g. Hypothyroidism (underactive thyroid)
    h. Rheumatic diseases (including rheumatoid arthritis, psoriatic arthritis,…)
    i. Other: ………………………………..

28. What is your highest degree?
    a. Primary education
    b. Professional secondary education
    c. Technical secondary education
    d. Artistic secondary education
    e. General secondary education
    f. Professional bachelor
    g. Academic bachelor
    h. Master
    i. PhD
    j. Other: ……….

29. What is your current professional status? (if you are currently not working because of pregnancy complications or pregnancy leave, please fill in the professional status you had before)
    a. Employee
        a. Are/were you working in healthcare?
    b. Self-employed
        a. Are/were you working in healthcare?
    c. Civil service employee
        a. Are/were you working in healthcare?
    d. Student
    e. Housewife
    f. Jobseeker/unemployed
    g. Incapacitated/disabled
    h. Other: ………

Coronavirus and your financial situation

30. Did the coronavirus affect your income?
    a. Yes
    b. No
    c. Not applicable

31. If yes: How did the coronavirus affect your income?
    a. Negatively
    b. Positively

32. If yes: To which extent did the coronavirus affect your income?
    a. Limited influence
    b. Large influence

SURVEY BREASTFEEDING

CORONAVIRUS AND BREASTFEEDING

1. Are you currently breastfeeding?
    a. Yes
    b. No

IF YES:

2. Please indicate the age of the baby that is currently breastfed.
    a. ≤ 6 weeks
    b. Between 6 weeks and 6 months
    c. > 6 months

3. Did the diet of your baby change due to the coronavirus?
    a. Yes
    b. No

4. If yes: please indicate which applies:
    a. I give/gave breastmilk (via breastfeeding or expressed breastmilk) more often as compared to before the outbreak of the coronavirus
        a. If yes, please indicate why. You can choose multiple answers.
            a. A healthcare professional advised me to breastfeed more frequently
            b. A family member / friend advised me to breastfeed more frequently
            c. I want(ed) to protect my baby against the coronavirus through breastmilk
            d. Due to the coronavirus I am/was at home with my baby more often
            e. Other: ……………………………………………………………………….

    b. I give/gave breastmilk (via breastfeeding or expressed breastmilk) less often as compared to before the outbreak of the coronavirus
        a. If yes, please indicate how this can be explained. You can choose multiple answers.

a. I don't/didn't want to infect my baby while breastfeeding
b. I am/was afraid that the virus would reach my baby through breastmilk
c. I feel/felt too sick to breastfeed
d. A healthcare professional advised me to stop breastfeeding
e. A family member / friend advised me to stop breastfeeding
f. I did not receive or find a reassuring answer to my questions about coronavirus and breastfeeding
g. Due to my illness, I use(d) a medicine that is incompatible with breastfeeding
h. Due to concerns for the virus, I do/did not produce enough milk anymore
i. Due to the coronavirus, I have/had to work (more) and could no longer combine it with breastfeeding
j. Due to the coronavirus, I do/did not receive sufficient support from a healthcare professional with regard to breastfeeding and/or breastfeeding related issues
k. Due to childcare responsibilities or circumstances in my home
l. Other: ……………………………………………………………………….

c. I entirely switched from breastfeeding to expressing breastmilk
d. Other: ……………………………………..

5. To what extent have you considered stopping breastfeeding/giving expressed milk due to the coronavirus?
   a. Not considered at all
   b. Barely considered
   c. Slightly considered
   d. Strongly considered

6. To what extent have you considered breastfeeding/giving expressed breastmilk for a longer period of time?
   a. Not considered at all
   b. Barely considered
   c. Slightly considered
   d. Strongly considered

7. Did you ever breastfeed before the coronavirus outbreak?
    a. Yes
    b. No

8. If yes: compared to with the previous time / times that you breastfed, to what extent has the coronavirus affected your experience of breastfeeding?
    a. No influence at all
    b. Rather no influence
    c. Rather large influence
    d. Large influence

9. Has the coronavirus pandemic affected how you interact with health services during the breastfeeding period?
    a. Yes
    b. No

10. If yes: please indicate how the coronavirus pandemic has affected your interaction with health services, and whether you receive(d) more, less or equal follow-up as before (more follow-up, less follow-up, no influence, not applicable)
    a. Follow-up by the general practitioner
    b. Follow-up by the midwife
    c. Follow-up by the obstetrician
    d. Follow-up by the paediatrician
    e. Follow-up by the specialist (other than obstetrician, paediatrician or GP)
    f. Follow-up by the lactation consultant
    g. Follow-up by a maternal or perinatal organization

11. Has the coronavirus pandemic affected the support you receive(d) during the breastfeeding period?
    a. Yes
    b. No

12. If yes: please indicate how the coronavirus pandemic affected the support you received, and whether you receive(d) more, less or equal support as before (more support, less support, no influence, not applicable)
    a. Support from maternity care services at home
    b. Support from other maternal or perinatal organizations
    c. Support from family
    d. Support from friends

IF NO:

13. Please indicate the current age of the baby that was breastfed in the last 3 months.
    a. ≤ 6 weeks
    b. Between 6 weeks and 6 months
    c. > 6 months

14. Did you stop breastfeeding due to the coronavirus?
    a. Yes
    b. No

15. If yes: please indicate why you stopped breastfeeding. You can choose multiple answers.
    a. I didn't want to infect my baby while breastfeeding
    b. I was afraid that the virus would reach my baby through breastmilk
    c. I felt too sick to breastfeed
    d. A healthcare professional advised me to stop breastfeeding
    e. A family member / friend advised me to stop breastfeeding
    f. I did not receive or find a reassuring answer to my questions about coronavirus and breastfeeding
    g. Due to my illness, I use(d) a medicine that is incompatible with breastfeeding
    h. Due to concerns for the virus, I did not produce enough milk anymore
    i. Due to the coronavirus, I had to work (more) and could no longer combine it with breastfeeding
    j. Due to the coronavirus, I did not receive sufficient support from a healthcare professional with regard to breastfeeding and/or breastfeeding related issues

k. Due to childcare responsibilities or circumstances in my home
l. Other: ……………………………………………………………….

16. Did you ever breastfeed before the coronavirus outbreak?
    a. Yes
    b. No

17. If yes: compared to the previous time / times that you breastfed, to what extent has the coronavirus affected your experience of breastfeeding?
    a. No influence at all
    b. Rather no influence
    c. Rather large influence
    d. Large influence

18. Did the coronavirus pandemic affect how you interact with health services during the breastfeeding period?
    a. Yes
    b. No

19. If yes: please indicate how the coronavirus pandemic affected how you deal with health services, and whether you received more, less or equal follow-up as before (more follow-up, less follow-up, no influence, not applicable)
    a. Follow-up by the general practitioner
    b. Follow-up by the midwife
    c. Follow-up by the obstetrician
    d. Follow-up by the paediatrician
    e. Follow-up by the specialist (other than obstetrician, paediatrician or GP)
    f. Follow-up by the lactation consultant
    g. Follow-up by a maternal or perinatal organization

20. Did the coronavirus pandemic affect the support you received during the breastfeeding period?
    a. Yes
    b. No

21. If yes: please indicate how the coronavirus pandemic affected the support you received, and whether you received more, less or equal support as before (more support, less support, no influence, not applicable)
    a. Support from maternity care services at home
    b. Support from other maternal and perinatal organizations
    c. Support from family
    d. Support from friends

CORONAVIRUS SYMPTOMS

22. Did you receive a test to determine coronavirus infection?
    a. Yes
    b. No

23. If yes: Did you receive a nose/throat swab?
    a. Yes
    b. No

24. If yes: What was the result of the test? If you received multiple tests, please indicate 'positive' if at least one of the test results was positive
    a. Positive (infected with the coronavirus)
    b. Negative (not infected with the coronavirus)
    c. I don't know (yet)

25. If yes: Did you receive a blood test?
    a. Yes
    b. No

26. If yes: What was the result of the test?
    a. Confirmation of a current coronavirus infection
    b. Confirmation of a previous coronavirus infection
    c. No coronavirus infection
    d. I don't know (yet)

27. If yes: Did you receive a CT-scan of the lungs to determine a coronavirus related pneumonia?
    a. Yes
    b. No

28. If yes: what was the result of the test?
    a. The scan determined a coronavirus infection
    b. The scan did not determine a coronavirus infection
    c. I don't know (yet)

PERSONAL BELIEFS ABOUT THE CORONAVIRUS AND COVID-19 VACCINES

Please indicate to which extent you agree with the following statements:

29. If a coronavirus vaccine was available, I would get the vaccine during breastfeeding.
    a. Strongly agree
    b. Agree
    c. Disagree
    d. Strongly disagree

30. If I was infected with coronavirus during breastfeeding, I would be willing to participating in a scientific study where experimental medicines are tested.
    a. Strongly agree
    b. Agree
    c. Disagree
    d. Strongly disagree

CORONAVIRUS AND RESTRICTED ACTIVITIES

31. Did you personally decide to restrict yourself to only essential journeys because you were breastfeeding during the corona pandemic on top of the measures imposed by the government?
    a. Yes
    b. No

32. Are your normal activities currently restricted compared to before the coronavirus period?
    a. Yes
    b. No

PERSONAL BACKGROUND

In this final part, we will ask you a few questions about yourself as a participant of this study.

About you

33. Year of birth?

34. Where do you live?

35. Do you currently have a partner?
    a. Yes
    b. No

36. Have you smoked in the past 4 weeks?
    c. Yes
    d. No

37. Do you have any chronic illnesses? (these are conditions that already existed before your pregnancy)?
    a. Yes
    b. No

38. If yes: what chronic illness do you have?
    a. Asthma
    b. Allergy
    c. Cardiovascular disease (including high blood pressure, high cholesterol, heart disease)
    d. Depression
    e. Diabetes
    f. Epilepsy
    g. Hypothyroidism (underactive thyroid)
    h. Rheumatic diseases (including rheumatoid arthritis, psoriatic arthritis)
    i. Other: ………………………………..

39. What is your highest degree?
    a. Primary education
    b. Professional secondary education
    c. Technical secondary education
    d. Artistic secondary education
    e. General secondary education
    f. Professional bachelor
    g. Academic bachelor
    h. Master
    i. PhD
    j. Other: ……….

40. What is your current professional status? (if you are currently not working because of maternity or parental leave, please fill in the professional status you had before)
    a. Employee
        1. Are/were you working in healthcare?
    b. Self-employed
        1. Are/were you working in healthcare?
    c. Civil service employee

1. Are/were you working in healthcare?

d. Student
e. Housewife
f. Jobseeker/unemployed
g. Incapacitated/disabled
h. Other: ………

Coronavirus and your financial situation

41. Did the coronavirus affect your income?
    a. Yes
    b. No
    c. Not applicable

42. If yes: How did the coronavirus affect your income?
    a. Negatively
    b. Positively

43. If yes: To which extent did the coronavirus affect your income?
    a. Limited influence
    b. Large influence

Table S1: Comparison of the socio-demographic characteristics of the participants with country-specific birthing population data.

	BELGIUM			NORWAY			THE NETHERLANDS			IRELAND			SWITZERLAND			UNITED KINGDOM		
	General[a]	*Pregnant (n=2754)*	*Lactation (n=4268)*	*General*[b]	*Pregnant (n=1344)*	*Lactation (n=1485)*	*General*[c]	*Pregnant (n=1173)*	*Lactation (n=1447)*	*General*[d]	*Pregnant (n=692)*	*Lactation (n=912)*	*General*[e]	*Pregnant (n=563)*	*Lactation (n=1193)*	*General*[f]	*Pregnant (n=135)*	*Lactation (n=97)*
**Maternal age (years)**																		
18-25	10.1	3.6	2.4	9.4	11.8	7.6	6.8	6.6	2.4	9.8	7.0	1.4	8.1	2.8	1.7	22.7	11.9	2.9
26-30	34.1	40.9	32.3	31.3	40.8	33.9	26.7	38.0	29.9	17.8	15.3	9.1	26.4	25.1	20.5	28.6	18.8	22.1
31-35	37.2	44.6	48.0	37.4	36.0	40.4	40.8	40.0	49.4	36.0	44.1	44.8	39.3	47.5	49.7	29.4	38.6	45.6
36-40	15.2	10.0	15.2	17.8	10.3	15.7	21.1	14.9	15.8	29.4	28.2	37.0	21.8	22.0	23.1	15.7	26.7	22.1
> 40	3.4	0.9	2.2	3.7	1.2	2.4	4.6	0.5	2.6	6.4	5.5	7.7	4.4	2.6	4.9	3.6	4.0	7.4
**Relationship status**																		
Partner	81.5	97.1	97.0	94.0	98.8	98.7	91.0	98.3	97.6	84.5	98.0	98.7	83.2	99.1	99.0	94.8	94.2	98.6
No partner	19.5	2.9	3.0	6.0	1.2	1.3	9.0	1.7	2.4	15.5	2.0	1.3	16.8	0.9	1.0	5.2	5.8	1.4
**Professional status**																		
Professionally active	75.3	94.5	91.4	77.9	90.8	88.8	82.1	92.7	93.3	73.0	88.1	90.0	83.0	91.0	81.7	72.8	83.5	90.0
**Education level**																		
Low	12.0	0.5	0.6	17.1	2.8	2.2	12.2	5.6	2.6	19.0	0.6	0.1	42.7	25.7	33.1	45.7	12.9	22.4
Medium	40.0	14.9	17.6	23.8	19.0	15.7	35.4	30.0	22.5	37.0	38.8	54.3	12.0	23.4	17.8	18.6	23.7	7.5
High	48.0	84.6	81.8	59.0	78.2	82.1	50.9	64.5	74.9	43.0	60.6	45.6	45.3	50.9	49.1	30.4	63.4	70.1
**Smoking in pregnancy**																		
Yes	6.0	2.4	N/A	2.1/1.6[g]	1.0	N/A	7.4	3.0	N/A	10.9	3.5	N/A	7.0	5.9	N/A	12.4	1.9	N/A
No	94.0	97.6	N/A	97.9/98.4[g]	99.0	N/A	92.6	97.0	N/A	89.1	96.5	N/A	93.0	94.1	N/A	87.6	98.1	N/A

Data are presented as %. N/A = not available. [a]**BELGIUM:** Statistics for the general birthing population were retrieved from Statbel (education level (25-44 years, 2019); professional status (25-49 years, 2016)), SPE (maternal age, 2019) and Sexpert (relationship status (18-39 years, 2013)). Age categories reported in the perinatal statistics report slightly differ from those reported in the table: <25 years, 25-29, 30-34, 35-39, >40. Smoking in pregnancy was investigated in the publication with PMID 31342605. [b]**NORWAY**: Statistics for the general birthing population in Norway were retrieved from the Medical Birth Registry of Norway in 2019 (age, relationship status and smoking) (www.mfr.no). Official statistics from the Norwegian Medical Birth Registry have a slightly different age categorization; 18 to 24 years, 25 to 29 years, 30 to 34 years, 35 to 39 years, 40 to 44 years. Numbers reported follow this categorization. Statistics on educational level (aged 20-49) and professional status (aged 25 – 39 years) were retrieved from Statistics Norway in 2019 (www.ssb.no). [c]**THE NETHERLANDS**: Statistics for the general birthing population in the Netherlands were retrieved from CBS StatLine, general statistics Netherlands (data for 2019) (maternal age, education level, professional status and relationship status (2017)); and from Trimbose Institute for mental health (data for 2018) (smoking). [d]**IRELAND**: Characteristics for the general birthing population in Ireland (age and professional status) were estimated from the Perinatal Statistics report 2016 of the Health Service Executive (HSE). Age categories reported in the perinatal statistics report differ slightly from those reported in the table: <24 years, 25-29, 30-34, 35-39, >40. Professionally active status were estimated from mother's socio-economic grouping. Statistics for smoking in pregnancy were retrieved from PMID 28190202. Statistics on relationship status and education level in the general population were estimated from data reported by the Central Statistics Office (www.statbank.cso.ie). Data on relationship status relate to the proportion of family units who were reported as 'one parent mother with children' in the 2016 census. Data on educational level relate to educational attainment among females aged 19-64 years (Q2 2019). [e]**SWITZERLAND:** Statistics for the general birthing population in Switzerland were retrieved from the Federal Statistical Office (FSO) in 2019 for maternal age, relationship status (aged 25-44 years), professional status (aged 25-54 years), and education level (aged 25–34 years) (https://www.bfs.admin.ch). Smoking in pregnancy in Switzerland has been monitored on behalf of FSO between 2011 and 2016 (https://www.infoset.ch/fr/tabac.html). [f]**UNITED KINGDOM:** Statistics for the general birthing population are retrieved from various datasets provided by: (i) the Office of National Statistics including Conception Statistics for England and Wales (maternal age and relationship status – data from 2018), Labour Market Statistics (educational attainment in men and women aged 16-49 – data from 2011, and professional status in women aged 16-64 – data from Jan to Jun 2020) and Adult Smoking Habits in England (women aged 16-49 – data from 2019); (ii) NHS Digital Hospital Episode Statistics NHS Maternity Statistics (smoking at booking appointment – data from 2018-19). [g]start of pregnancy / end of pregnancy.

Table S2: Country-specific results of pregnant and breastfeeding women's perceptions about the coronavirus.

**Statements**	**(Strongly) agree**							**(Strongly) disagree**						
	Total	Belgium	Ireland	Norway	Switzerland	NL	UK	Total	Belgium	Ireland	Norway	Switzerland	NL	UK
Q1. «I believe that a coronavirus infection during pregnancy can affect the development of the unborn child» (n=6420)	**53.9% (3460)**	**62.8% (1729)**	**51.7% (320)**	32.8% (441)	**53.4% (275)**	**59.1% (634)**	**53.0% (61)**	46.1% (2960)	37.2% (1025)	48.3% (299)	**67.2% (903)**	46.6% (240)	40.9% (439)	47.0% (54)
Q2. «I believe that a coronavirus infection which seriously affects the health of the mother can affect the development of the unborn child» (n= 6420)	**86.0% (5524)**	**92.1% (2537)**	**82.9% (513)**	**71.1% (956)**	**83.9% (432)**	**91.5% (982)**	**90.4% (104)**	14.0% (896)	7.9% (217)	17.1% (106)	28.9% (388)	16.1% (83)	8.5% (91)	9.6% (11)
Q3. «I would consider a termination of pregnancy in case of a coronavirus infection during the first pregnancy trimester (n= 6420)	3.9% (252)	4.5% (123)	3.1% (19)	5.0% (67)	3.7% (19)	2.0% (21)	2.6% (3)	**96.1% (6168)**	**95.5% (2631)**	**96.9% (600)**	**95.0% (1277)**	**96.3% (496)**	**98.0% (1052)**	**97.4% (112)**
Q4. «If I was infected with coronavirus during pregnancy, I would be willing to participating in a scientific study where experimental medicines are tested» (n=6420)	14.5% (930)	9.6% (264)	25.2% (156)	24.9% (335)	13.6% (70)	7.3% (78)	23.5% (27)	**85.5% (5490)**	**90.4% (2490)**	**74.8% (463)**	**75.1% (1009)**	**86.4% (445)**	**92.7% (995)**	**76.5% (88)**
Q5. «If I was infected with coronavirus during breastfeeding, I would be willing to participating in a scientific study where experimental medicines are tested» (n=8980)	18.8% (1687)	12.3% (524)	28.4% (227)	39.5% (587)	14.9% (154)	13.2% (174)	26.6% (21)	**81.2% (7293)**	**87.7% (3744)**	**71.6% (572)**	**60.5% (898)**	**85.1% (881)**	**86.8% (1140)**	**73.4% (58)**

Results are expressed as % (n). Questions Q1-Q4 were part of the survey for pregnant women, while question Q5 was part of the breastfeeding survey. For each statement and each individual country, the results of the most commonly reported answer are highlighted in bold.

Table S3: Country-specific results of COVID-19 vaccine willingness during pregnancy and breastfeeding according to professional status.

	Total	Belgium	Ireland	Norway	Switzerland	NL	UK
**COVID-19 vaccine willingness in pregnancy**							
Professionally active, not in healthcare	64.7% (2265)	80.4% (1293)	50.3% (173)	57.5% (454)	30.9% (79)	53.4% (238)	51.9% (28)
Professionally active, in healthcare	62.3% (1215)	76.7% (736)	57.0% (73)	52.6% (206)	32.5% (40)	44.7% (142)	60.0% (18)
Not professionally active	50.2% (228)	63.2% (96)	53.1% (34)	50.8% (62)	13.2% (5)	37.7% (23)	47.1% (8)
**COVID-19 vaccine willingness during breastfeeding**							
Professionally active, not in healthcare	72.1% (3487)	80.5% (1951)	71.4% (304)	69.3% (630)	41.4% (193)	65.8% (371)	80.9% (38)
Professionally active in healthcare	70.9% (1857)	80.5% (1138)	58.2% (114)	68.1% (263)	38.8% (76)	61.6% (253)	86.7% (13)
Not professionally active	56.9% (470)	64.9% (237)	71.0% (49)	59.1% (97)	34.7% (52)	40.8% (29)	85.7% (6)

Results are expressed as % (n). The percentages represent the number of pregnant or breastfeeding women (strongly) agreeing on the statements: "if a coronavirus vaccine was available, I would get the vaccine during *pregnancy* or *breastfeeding*", respectively. The statements were rated on a 4-point Likert scale ranging from (strongly) agree to (strongly) disagree. Only the percentages of women (strongly) agreeing are shown in the table.

Table S4: Overview of representative statements of participants.

**Pregnancy /breastfeeding experience**	"Due to the corona measures, it **felt like I was pregnant alone** instead of my partner and I expecting a baby. I miss his support and I miss it to experience the pregnancy together. I am angry that he is considered redundant with regard to the care for our unborn child." *(the Netherlands)*<br><br>"I find the **coronavirus less exciting during breastfeeding than during pregnancy**. What is the impact of corona on your unborn child? And what if you have COVID-19 during the delivery, it's a hassle with wearing masks and being healthy enough to puff and squeeze." *(the Netherlands)*<br><br>"I tested positive for the coronavirus in April, in the 8th month of pregnancy. This had no consequences for my baby. Antibodies were found in the blood of the placenta at birth. This is why **I am not worried about breastfeeding**." *(Switzerland)*<br><br>"My husband had to leave immediately after the birth of our child and wasn't allowed back until 4 days later when I was discharged, so **he missed out on 4 days of bonding with our daughter**." *(United Kingdom)*<br><br>"I **have found pregnancy during the pandemic very lonely** and thought I would get a beautiful pregnancy to share with family, I ended up having scans alone and labouring alone until I was 4cm. Nothing was what I expected and even now I can't have people visit my newborn." *(United Kingdom)*<br><br>"Less peace and quiet around me when breastfeeding and caring for my child because I have had 2 school children at home, in addition to one kindergarten child (closed kindergartens and schools). Less peace and quiet in everyday life around the baby due to follow-up of homework (home-schooling), as well as activation of a busy 3-year-old all day. **Less support from health care workers because the maternal and child health center has worked differently, as there have been no maternity groups to share experiences with and get support from**. Less support from other adults, compared to my other maternity periods, as we have not been in contact with grandparents, aunts and uncles either. **More isolated, more frustration, and the feeling of not having time for breastfeeding, the baby, and the other children's needs**." *(Norway)*<br><br>"I felt **less worried that people would find out**. I didn't have to think about making up excuses for meeting people." *(Ireland)*

Breastfeeding practices	“Thanks to the coronavirus, I work from home. During the previous breastfeeding period, I found pumping challenging and therefore I stopped earlier than I wanted. Now, **I can give my child more often the breast which gives me less stress. I also want to protect my child,** so I don’t want to stop.” *(the Netherlands)*<br><br>“I have been breastfeeding longer. Especially because I became ill myself and might have had COVID-19, so there was a chance that **my child could get antibodies from my breastmilk and that I would protect him through my breastmilk**.” *(the Netherlands)*<br><br>“I did not want to **infect my child during breastfeeding.** I was afraid that the virus could be transmitted to my child through breast milk. I did not receive or find reassuring answers to my questions about coronavirus and breastfeeding.” *(Switzerland)*<br><br>“There is **no reason why you should stop [breastfeeding]** because of the pandemic, in fact it is the best we can do to protect our children **with the antibodies we pass on**, that can help our children’s immune systems to develop and fight off other viruses and infections.” *(United Kingdom)*<br><br>“The pandemic was a reminder of the importance of breastfeeding, including in terms of antibodies and protection**. Breastfeeding contributes to security in an unsafe time.**” *(Norway)*<br><br>“I became corona-infected myself. Thought it was good that I breastfed my baby due to possible antibodies, but thought it was challenging to be so close to my baby when I also knew I was contagious. **Due to isolation, I waited a little longer before I introduced solid foods.**” *(Norway)*<br><br>“I probably would not have persisted with breastfeeding only for the lockdown. Lockdown meant I had no visitors or anywhere to go so it **allowed me time to dedicate to breastfeeding** which was nearly all of my time when my son was small. I **would have liked to attend a breastfeeding support group** in my area to meet new mothers close by.” *(Ireland)*

Access to health services / support in the perinatal period	“I have experienced several serious health issues during this pregnancy, but mostly I was left on my own, with only a single check-up so **several issues have been detected very late**. For example, I already had diabetes symptoms for months before I could be tested and then it turned out that the **baby was already in the 92nd percentile in terms of growth**. **Nobody feels responsible** and **I have no clear contact person**.” *(the Netherlands)*<br><br>“I **truly missed the maternity visits** and the interest of family and friends.” *(the Netherlands)*<br><br>“My baby suffered from bronchiolitis at 3 weeks of age. **Coming home was difficult and there was not enough medical support**. The paediatrician didn't have room and I felt helpless with my baby's respiratory distress due to his cold.” *(Switzerland)*<br><br>“I have found that with a **lack of available support from family and friends**, simple things that I could have had help with I haven’t been able to, so my mental health has taken a nosedive. Moreover, my baby’s health has been ignored as he hasn’t gained enough weight, but until 9 weeks I was advised on the phone to keep breastfeeding. His weight was so low he’d dropped off of the chart and no one was available to support us. **New mums have been forgotten**!” *(United Kingdom)*<br><br>“The child's father has not been allowed to join either the ultrasound examinations, birth preparatory courses or visits at the midwifes. I have felt quite alone. I’m struggling with pelvic girdle pain but have not been able to participate in pregnancy swimming or get an appointment with a physiotherapist during the corona pandemic due to **everything being canceled and closed**.” *(Norway)*<br><br>“**My partner was not allowed to participate when I had the egg insertion (ART) nor early ultrasound to confirm pregnancy**.” *(Norway)*<br><br>“I found the whole **postpartum experience very isolating** and that the whole focus was on pregnancy and birth but **no information or support once you left the hospital**.” *(Ireland)*

Figure S2: Overview of routine antenatal care in participating countries along with the COVID-19 regulations regarding maternity care.

1. Belgium

Routine antenatal care includes at least 7 recommended prenatal visits, the exact number depending on women's parity (7 for multiparae, 10 for primiparae). The check-ups take place with the obstetrician and/or GP or midwife. The first check-up takes place around 8-12 weeks, including the mapping of individual risk factors. In Belgium, 3 screening ultrasounds are recommended during pregnancy (at 11-14w; 18-23w; 28-34w); in case of complications additional ultrasounds can be performed. High-risk pregnancies are more frequently followed-up. At the time of study execution, **due to COVID-19 regulations**, restrictions in the presence of the partner during prenatal visits, ultrasounds and the delivery were possible depending on the individual hospital's policy.

2. Norway

Routine antenatal care: Routine antenatal care includes 8 standard maternity check-ups with the midwife or GP. The first check-up is around gestational week 10 (incl. mapping of risk factors). Moreover, all women are offered one routine ultrasound examination around gestational week 17-19 to determine expected delivery date. Women with risk pregnancies have more frequent and detailed follow-ups.

COVID-19 regulations and maternity care: the major change was that there were restrictions to what the father could participate in. The father could be present under the standard ultrasound (US) at week 18, but not at other check-ups at the hospitals, and he could only be present during active delivery.

3. Ireland

Routine antenatal care: Under the Maternity and Infant Care Scheme, all pregnant women resident in Ireland are entitled to free public maternity care. Most women receive combined antenatal care from their GP and an obstetrician and/or midwife in a consultant-led maternity unit. Other women receive all of their antenatal care from a maternity unit. Antenatal care may also be shared by a GP and midwives in one of Ireland's two midwifery-led units. A small number of women choose to birth at home and will receive antenatal care from a self-employed community midwife in combination with their GP.

The first visit to the maternity unit takes place between 8- and 12-weeks' gestation and includes an assessment of the woman's obstetric and medical history (commonly known as the 'booking visit') and an ultrasound dating scan. An additional anatomy scan may be offered between 20-22 weeks of pregnancy. Women will attend their GP between 5 and 6 times in pregnancy, and their maternity care provider at least 6 times. Women with complex obstetric or medical histories will be seen more frequently during pregnancy and additional scans may be offered. Additional ultrasound scans, non-invasive and invasive testing may also be obtained through private maternity services. **During the COVID-19 pandemic**, partners could not accompany women to antenatal and ultrasound appointments and could only accompany their partner at active delivery.

4. United Kingdom (UK)

In the UK, **routine antenatal care** begins when women notify their general practitioner (family doctor) of their pregnancy. Pregnant women are subsequently seen by their community midwife between eight- and twelve-weeks gestational age. At this appointment, the midwife will take a complete medical and obstetric history and provide information about the antenatal care plan. Women with chronic medical conditions, a history of pregnancy complications or adverse pregnancy outcomes may be referred to an obstetrician for the remainder of their antenatal care. Women considered to be at low risk of complications or

adverse outcome will be cared for by community midwives and be seen at least monthly for the remainder of their pregnancy. Pregnant women in the UK are given two routine ultrasound scans, the first is performed between eight- and 14-weeks gestational age to identify the expected due date and to screen for some genetic conditions (Down's, Edwards' and Patau's). The second is performed between 18- and 20-weeks gestational age to screen for developmental anomalies. Additional ultrasound scans may be required depending on the course of the pregnancy. **During the first wave of the COVID-19 pandemic**, birth partners were not permitted to attend routine antenatal check-ups or ultrasound scans. In some hospitals, partners were not permitted to support their partners in hospital during the early stages of labour but could attend the active delivery. Some hospitals also did not permit partners to remain on the maternity ward after the delivery.

5. Switzerland

Routine antenatal care includes 8 standard maternity check-ups with the obstetrician and/or midwife. The first visit takes place around 8-10 weeks and includes the mapping of individual risk factors (medical history, examination, biology). Then, a monthly follow-up is the basis, but high-risk pregnancies receive generally more examinations. The first ultrasound takes place between 11 and 14 weeks and includes a risk assessment for Down Syndrome, as well as non-invasive or invasive testing if necessary. A second ultrasound is performed around week 22, and additional US are planned based on individual risk factors. **During the first wave of the COVID-19 pandemic**, partners were not allowed during prenatal check-ups and ultrasounds, but they could attend the active delivery.

6. The Netherlands

Routine antenatal care includes 12 or 13 standard maternity check-ups with the obstetrician and/or midwife. The intake visit takes place around 8-10 weeks. A vitality ultrasound is performed at 10-12 weeks and an extensive ultrasound at 20 weeks. **During the first wave of the pandemic**, partners were not allowed during prenatal check-ups and ultrasounds, but could be present during delivery. The intake was done by phone, the vitality ultrasound was skipped and the prenatal check-ups at 23 and 33 weeks were dropped. In May 2020, the measures were eased: partners were reallowed at all appointments and the original ultrasound and check-up scheme were reintroduced, except for the fact that the intake and the prenatal visits at 23 and 33 weeks were done by phone.

Table S5: Country-specific results of pregnant women's self-reported impact of the pandemic on access to health services.

Healthcare professional	More follow-up					Less follow-up					No influence				
	**Ireland**	**Norway**	**Switzerland**	**NL**	**UK**	**Ireland**	**Norway**	**Switzerland**	**NL**	**UK**	**Ireland**	**Norway**	**Switzerland**	**NL**	**UK**
Obstetrician (n=1184)	8.8% (36)	3.7% (7)	4.9% (12)	5.6% (15)	2.7% (2)	42.4% (174)	43.5% (83)	21.7% (53)	**62.0%** (165)	**54.8%** (40)	**48.8%** (200)	**52.9%** (101)	**73.4%** (179)	32.3% (86)	42.5% (31)
Midwife (n=1916)	9.2% (38)	4.3% (27)	5.5% (9)	2.6% (16)	2.9% (3)	42.7% (177)	**71.4%** (450)	38.0% (62)	**88.6%** (536)	**63.1%** (65)	**48.2%** (200)	24.3% (153)	**56.4%** (92)	8.8% (53)	34.0% (35)
GP (n=1510)	8.5% (39)	6.3% (38)	5.8% (8)	5.0% (12)	1.4% (1)	**54.0%** (247)	**56.4%** (341)	21.2% (29)	44.2% (106)	**57.7%** (41)	37.4% (171)	37.4% (226)	**73.0%** (100)	**50.8%** (122)	40.8% (29)
Medical specialist (n=858)	9.3% (24)	7.3% (19)	8.5% (10)	4.9% (8)	8.6% (5)	42.0% (108)	39.5% (103)	16.1% (19)	33.5% (55)	**48.3%** (28)	**48.6%** (125)	**53.3%** (139)	**75.4%** (89)	**61.6%** (101)	43.1% (25)

Results are expressed as % (absolute numbers). Percentages were calculated for each country and type of healthcare professional using as denominator the total number of women who reported that their access to health services had been affected by the pandemic (n=2266) and who indicated to be counseled by this specific type of healthcare professional during pregnancy. The highest percentage for each country and type of healthcare professional is highlighted in bold. GP = general practitioner.

Table S6: Country-specific results of breastfeeding women's self-reported impact of the pandemic on access to health services.

Healthcare professional	More follow-up					Less follow-up					No influence				
	**Ireland**	**Norway**	**Switzerland**	**NL**	**UK**	**Ireland**	**Norway**	**Switzerland**	**NL**	**UK**	**Ireland**	**Norway**	**Switzerland**	**NL**	**UK**
GP (n=2076)	3.4% (23)	2.7% (23)	7.6% (20)	0.9% (2)	5.4% (3)	**69.2%** (467)	**72.7%** (619)	43.7% (115)	**60.9%** (140)	**71.4%** (40)	27.4% (185)	24.6% (210)	**48.7%** (128)	38.3% (88)	23.2% (13)
Midwife (n=1831)	7.6% (42)	1.9% (11)	9.2% (30)	3.0% (10)	4.8% (2)	**61.3%** (338)	**72.1%** (416)	**53.2%** (173)	**76.8%** (258)	**50.0%** (21)	31.0% (171)	26.0% (150)	37.5% (122)	20.2% (68)	45.2% (19)
Obstetrician (n=1357)	2.6% (12)	1.1% (4)	7.2% (23)	0.5% (1)	0.0% (0)	46.2% (215)	43.6% (153)	30.0% (96)	**56.8%** (113)	**50.0%** (11)	**51.2%** (238)	**55.3%** (194)	**62.8%** (201)	42.7% (85)	**50.0%** (11)
Paediatrician (n=1606)	4.2% (19)	1.4% (8)	7.5% (27)	1.6% (3)	0.0% (0)	42.4% (193)	42.2% (241)	23.3% (84)	39.4% (76)	**66.7%** (18)	**53.4%** (243)	**56.4%** (322)	**69.2%** (249)	**59.1%** (114)	33.3% (9)
Medical specialist (n=1014)	22.5% (9)	2.5% (6)	6.7% (15)	0.0% (0)	0.0% (0)	**60.0%** (240)	**51.9%** (124)	27.4% (61)	29.5% (36)	**70.0%** (21)	37.8% (151)	45.6% (109)	**65.9%** (147)	**70.5%** (86)	30.0% (9)
Lactation consultant (n=1782)	5.6% (27)	1.1% (8)	4.7% (12)	2.7% (7)	3.4% (1)	**69.6%** (334)	**77.1%** (584)	42.5% (108)	**68.7%** (180)	**82.8%** (24)	24.8% (119)	21.8% (165)	**52.8%** (134)	28.6% (75)	13.8% (4)
Perinatal organization (n=1127)	2.7% (12)	-	7.1% (19)	1.6% (6)	0.0% (0)	**70.8%** (318)	-	**58.6%** (157)	**81.8%** (311)	**83.3%** (25)	26.5% (119)	-	34.3% (92)	16.6% (63)	16.7% (5)

Results are expressed as % (absolute numbers). Percentages were calculated for each country and type of healthcare professional using as denominator the total number of women who reported that their access to health services had been affected by the pandemic (n=2644) and who indicated to be counseled by this specific type of healthcare professional during breastfeeding. The highest percentage for each country and type of healthcare professional is highlighted in bold. The variable 'perinatal organization' was not included in the Norwegian breastfeeding survey. GP = general practitioner.

Table S7: Country-specific results of breastfeeding women's self-reported impact of the pandemic on support during breastfeeding.

Support Provided by	More Support					Less Support					No Influence				
	**Ireland**	**Norway**	**Switzerland**	**NL**	**UK**	**Ireland**	**Norway**	**Switzerland**	**NL**	**UK**	**Ireland**	**Norway**	**Switzerland**	**NL**	**UK**
Maternity care services at home (n=1680)	2.8% (15)	1.5% (8)	4.9% (16)	6.5% (16)	3.9% (2)	**83.5%** (444)	**88.2%** (462)	**73.2%** (238)	33.5% (83)	**90.2%** (46)	13.7% (73)	10.3% (54)	21.8% (71)	**60.1%** (149)	5.9% (3)
Perinatal organization (n=1447)	2.6% (13)	3.7% (11)	3.4% (10)	3.0% (9)	4.2% (2)	**85.8%** (435)	**81.3%** (244)	**67.0%** (197)	**82.9%** (247)	**87.5%** (42)	11.6% (59)	15.0% (45)	29.6% (87)	14.1% (42)	8.3% (4)
Family (n=1925)	10.7% (64)	12.5% (74)	15.0% (56)	10.0% (30)	0.0% (0)	**80.2%** (481)	**67.0%** (396)	**71.0%** (265)	**71.5%** (216)	**96.6%** (57)	9.2% (55)	20.5% (121)	13.9% (52)	18.5% (56)	3.4% (2)
Friends (n=1887)	4.8% (29)	5.3% (30)	9.4% (34)	2.7% (8)	0.0% (0)	**86.0%** (514)	**79.4%** (452)	**76.5%** (277)	**78.8%** (234)	**95.1%** (58)	9.2% (55)	15.3% (87)	14.1% (51)	18.5% (55)	4.9% (3)

Results are expressed as % (absolute numbers). Percentages were calculated for each country and type of support using as denominator the total number of women who reported that the support they received during the breastfeeding period was affected by the pandemic (n=2008) and who indicated that the type of support was applicable to them during breastfeeding. The highest percentage for each country and type of support is highlighted in bold.

Table S8: Imposed regulations per country during the first wave of the COVID-19 pandemic.

Country	First detected case	Start date of lockdown measures	End date of lockdown measures						
			Schools	Health services	Travel	Retail/shops	Bars & restaurants	Sport & facilities	Events
**Belgium**	04/02	13/03	18/05 (primary & secondary school)	Elective procedures and appointments were postponed. Visiting restrictions in hospitals and nursing homes	08/06 (in Belgium) 15/06 (Europe)	Most shops reopened 11/05 (supermarkets remained open)	08/06	04/05 (outdoor, max. 2 people) 18/05 (outdoor in team, no contact) 08/06 (indoor)	01/07 (max 200 indoor, max 400 outdoor)
**Norway**	26/02	01/03 (for HCP) 12/03 (for society)	20/4 (Kindergartens) 27/4 (Primary schools 1st to 4th grade) 11/5 (all primary and secondary schools)	Healthcare services didn't close 06/05 (some visitation at nursing homes) 20/04 (psychologists, physio-therapists)	Still applied for some countries 15/06 travel to Nordic countries (still restrictions to most of Sweden)	Shops remained open	01/06 (bars, cafeterias not serving food)	07/05 (sport-activities max 20) 15/06 (pools) 15/06 (sport activities, gyms)	07/05 private max 20, organizer max 50 15/06 organizer max 200
**Switzerland**	25/02	20/03	11/05 (primary) 8/06 (secondary)	Health care services didn't close	15/06 (quarantine for 2 weeks post-return is required for some countries)	27/04 for DIY shops, garden store and flower shops, 11/05 for most shops	11/05 (restaurants with max 4 people sitting) 06/06	11/05 (sport without contact, group of max 5 people) 22/06 (allowing contact sports)	06/06 max 30 people in outdoor space. 22/06 max 1000 in outdoor space
**The Netherlands**	27/02	15/03	11/05 (primary) 2/06 (secondary)	Around 1/05 (varies per hospital)	Still applied for some countries	Most shops remained open	1/06	11/5: tennis, football 1/7: gym, sauna	1/07 (indoor max 100, outdoor max 250)
**Ireland**	29/02	12/03	29/06	Healthcare services did not close. Elective procedures and out-patient appointments were rescheduled or changed to telehealth. Visiting restrictions were implemented in hospitals at varying time points	National travel restrictions ended 28/06. However, some international travel restrictions were still in place	Most restrictions lifted 07/06. Remaining restrictions lifted 28/06	Restrictions on restaurants lifted 07/06. Restrictions on bars remained in place	28/06	28/06: up to 50 people indoors and up to 200 people outdoors
**UK**	31/01	23/03 (national lockdown established enforceable by law)	NA (have been open for children of key workers throughout)	NA (general practice still remote and hospitals/dentists are only operating essential services)	10/07 (for some countries, other countries require quarantine for 2 weeks post-return)	15/06 (requirement for shoppers to wear masks)	04/07 (open where social distancing 2m can be maintained)	25/07	11/07 (outdoor only)

Figure S3: Overview of the COVID-19 cases and death notification rates per country during the first wave of the pandemic.
Source: European Centre for Disease Prevention and Control. Report: Resurgence of reported cases of COVID-19 in the EU/EEA, the UK and EU candidate and potential candidate countries (2 July 2020) (https://www.ecdc.europa.eu/sites/default/files/documents/RRA-Resurgence-of-reported-cases-of-COVID-19-in-the-EU-EEA.pdf).

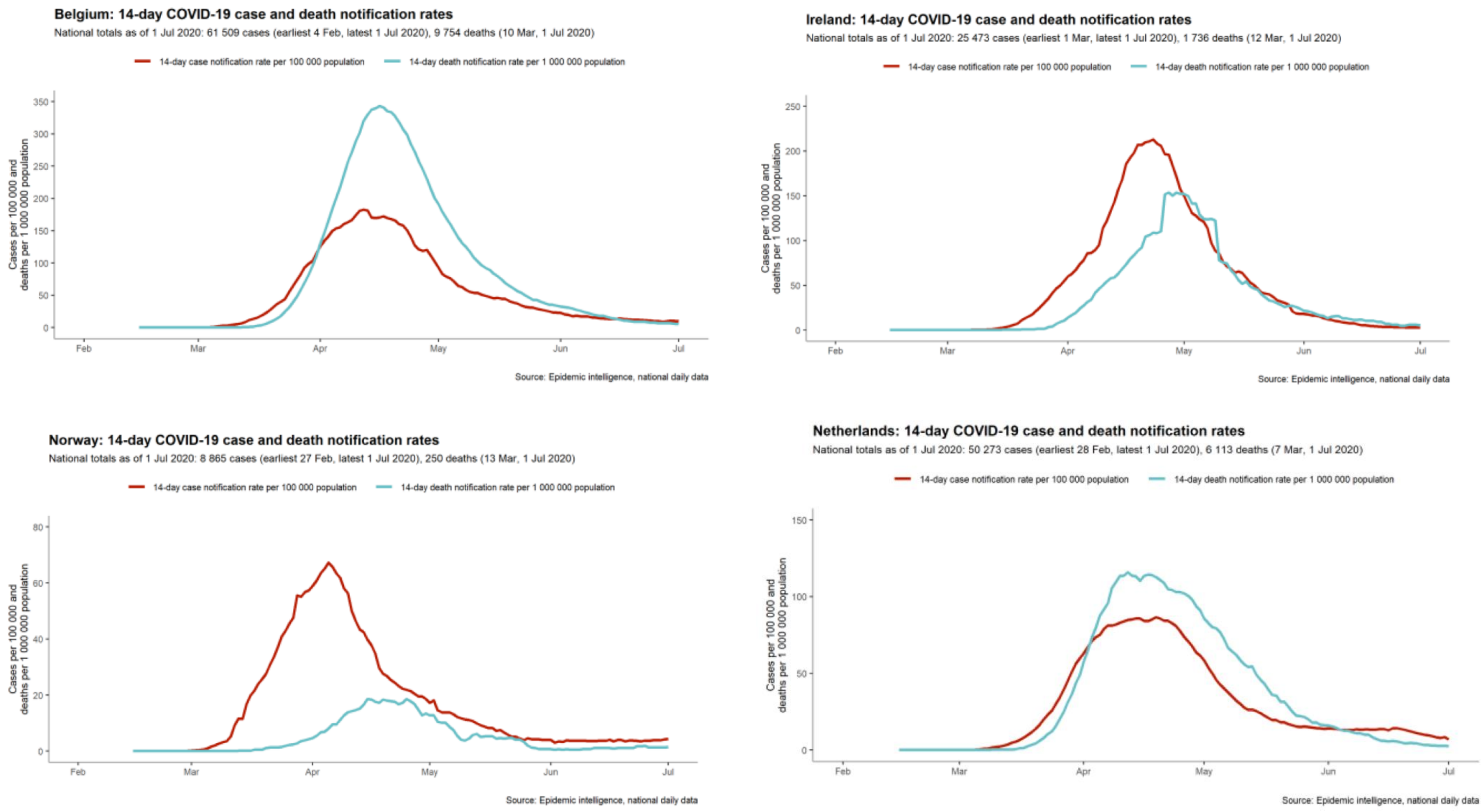

United Kingdom: 14-day COVID-19 case and death notification rates

National totals as of 1 Jul 2020: 312 654 cases (earliest 31 Jan, latest 1 Jul 2020), 43 730 deaths (7 Mar, 1 Jul 2020)

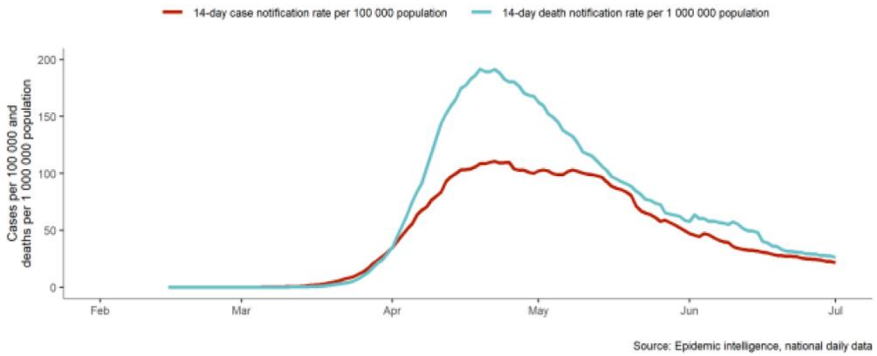

Supplement: Supplementary file 1 [file ijerph-18-03367-s001.pdf]
